# High Preservation of CpG Cytosine Methylation Patterns at Imprinted Gene Loci in Liver and Brain of Aged Mice

**DOI:** 10.1371/journal.pone.0073496

**Published:** 2013-09-09

**Authors:** Silvia Gravina, Martijn E. T. Dollé, Tao Wang, Harry van Steeg, Paul Hasty, Jan Hoeijmakers, Jan Vijg

**Affiliations:** 1 Department of Genetics, Albert Einstein College of Medicine, New York, New York, United States of America; 2 National Institute of Public Health and the Environment, Bilthoven, The Netherlands; 3 Department of Epidemiology and Population Health, Albert Einstein College of Medicine, New York, New York, United States of America; 4 Department of Molecular Medicine and Institute of Biotechnology, University of Texas Health Science Center, San Antonio, Texas, United States of America; 5 MGC Department of Genetics, CBG Cancer Genomics Center, Erasmus Medical Center, Rotterdam, The Netherlands; University of Texas MD Anderson Cancer Center, United States of America

## Abstract

A gradual loss of the correct patterning of 5-methyl cytosine marks in gene promoter regions has been implicated in aging and age-related diseases, most notably cancer. While a number of studies have examined DNA methylation in aging, there is no consensus on the magnitude of the effects, particularly at imprinted loci. Imprinted genes are likely candidate to undergo age-related changes because of their demonstrated plasticity *in utero*, for example, in response to environmental cues. Here we quantitatively analyzed a total of 100 individual CpG sites in promoter regions of 11 imprinted and non-imprinted genes in liver and cerebral cortex of young and old mice using mass spectrometry. The results indicate a remarkably high preservation of methylation marks during the aging process in both organs. To test if increased genotoxic stress associated with premature aging would destabilize DNA methylation we analyzed two DNA repair defective mouse models showing a host of premature aging symptoms in liver and brain. However, also in these animals, at the end of their life span, we found a similarly high preservation of DNA methylation marks. We conclude that patterns of DNA methylation in gene promoters of imprinted genes are surprisingly stable over time in normal, postmitotic tissues and that the multiple documented changes with age are likely to involve exceptions to this pattern, possibly associated with specific cellular responses to age-related changes other than genotoxic stress.

## Introduction

Aging is a process of time-dependent functional decline and increased incidence of disease occurring in all higher organisms. The molecular changes that underlie aging remain poorly understood, but one factor thought to play a major role is a general decline in the fidelity of information transfer, from DNA to RNA and protein, leading to what is sometimes referred to as epigenetic drift [1], [2]. Epigenetic drift can be defined as a gradual relaxation of originally tightly controlled cell and tissue-specific molecular phenotypes resulting in increased variation within the organism. Evidence for such a process originally came from observations of the loss of organ-specific gene silencing [3], but were more recently confirmed by reports showing increased epigenetic variation between monozygotic twins during aging [4], increased cell-to-cell variation in transcript levels among somatic cells in aged mouse heart [5] and a relaxation of genetic control of gene expression in old mice [6].

Probably the best-documented example of epigenetic modification is DNA methylation, which plays important roles in gene silencing and genome stability. A DNA methylation mark consists of a covalent addition of a methyl group to cytosine, i.e., 5-methylcytosine. In differentiated cells 5-methylcytosine occurs virtually only at CpG’s where it has been found in about 65% of all CpGs [7]. DNA methylation status is known to undergo changes with age in a subset of genes in a tissue-specific fashion [8]–[11] and *de novo* DNA methylation of promoter regions of tumor suppressor genes and their transcriptional silencing is a frequent alteration in tumors that possibly relates to the increased incidence of cancer with age [12].

Good candidates to undergo age-related changes in DNA methylation are imprinted genes, which are expressed from only one of the two parental alleles in a manner that depends on the parental origin of the allele. The parental difference is dictated by allele-specific methylation profiles that are faithfully transmitted to daughter cells during somatic cell division. While thought to be perpetuated throughout life in all tissues, environmental influences that impair the fidelity of DNA methylation of imprinted genes during maintenance *in utero* are well documented [13]–[15].

The question arises if age-related DNA methylation changes in promoter regions of imprinted genes occur randomly, affecting individual CpG sites, or are possibly related to systemic responses, for example, DNA damage responses, inflammation or overall hormonal and concomitant metabolic changes occurring with aging. To examine this in some more detail we quantitatively analyzed DNA methylation at CpG sites in the promoter regions of several randomly chosen imprinted genes in cerebral cortex and liver from young and old mice. For comparison we included two DNA repair defective mouse models, showing a range of premature aging symptoms as well as a significantly shorter life span. The results indicate exceptionally stable DNA methylation marks during aging or premature aging in the genes and organs analyzed.

## Methods

### Animals and Tissue Collection

Young adult (6 months) and old (27 months) C57BL/6 mice were obtained from the National Institute on Aging (NIA) and kept for at least two weeks in the animal facilities of the Albert Einstein College of Medicine, Bronx, New York. The *Ercc-/d7* mice (14 weeks) and *Ku80^−/−^* mice (10 months), both in C57BL/6-FVB F1 hybrid background, were kept in the animal facilities of the RIVM in Bilthoven, The Netherlands, together with their respective, litter mate, wild type controls. They were kept under specific pathogen-free (SPF) conditions and monitored for infections every 3 months. Experiments were performed with five animals for each group. Animals were killed by cervical dislocation. All surgical procedures and experimental manipulations were approved by the Ethics Committee for Animal Experiments in the Department of Genetics at the Albert Einstein College of Medicine in New York and the RIVM in Bilthoven. Experiments were conducted under the control of the Guidelines for Animal Experimentation.

### Genomic DNA Extraction

DNA from fresh or frozen tissues was isolated by phenol/chloroform extraction, as described [16].

### DNA Methylation Assay

For DNA methylation assay sodium bisulfite treatment was performed on 800 ng of DNA using the EZ DNA Methylation-Direct™ Kit (Zymo Research, CA, USA) following the manufacturer’s standard protocol. DNA methylation analysis was conducted following bisulfite-PCR amplification using the Sequenom EpiTYPER system (Sequenom Inc, CA, USA) as described elsewhere [17]. This technique employs base-specific cleavage followed by MALDI-TOF mass spectrometry in which the size ratio of the cleaved products provides quantitative methylation estimates for CpG sites within a target region [18]. Each EpiTYPER run was performed in duplicate. To select imprinted target genes we consulted a comprehensive database for imprinted genes available at http://igc.otago.ac.nz/home.html. Bisulfite primer sequences were designed using the EpiDesigner software or taken from the Mouse Standard EpiPanel (http://www.sequenom.com/getdoc/913a6da8-b7bf-40d6-aeab-c827f5a8e3b8/mouse_epipanel), which lists primer sequences validated by Sequenom. A representative example of a targeted region in the Gad1 promoter, is shown in Fig. S1 in File S1. The forward primers were tagged with a 10-mer (5′-AGGAAGAGAG-3′) to balance the PCR, and the reverse primers contained a T7-promoter tag (5′-CAGTAATACGACT CACTATAGGGAGAAGGCT-3′) for *in vitro* transcription. Bisulfite-PCR amplification was conducted using the HotStarTaq Master Mix Kit (Qiagen, USA) under the following conditions: 95°C for 15 minutes, then 35 cycles at 94°C for 30 seconds, Tm for 30 seconds and 72°C for 30 seconds, followed by 72°C for 10 minutes for the final extension. Results were analyzed using the EpiTYPER software.

### Statistical Analysis

We performed the Wilcoxon test to evaluate CpG sites differing in methylation between groups. Bonferroni correction was applied to the Ube3a gene (CpG 10). Levene’s test for homogeneity of variance was applied to test individual variation between groups. T test was applied to compare the differences in average DNA methylation between groups. All p-values can be found in Tables S1–8 in File S1.

## Results

### DNA Methylation Analysis in Liver and Cerebral Cortex from Young and Old Mice

In this study we chose cerebral cortex and liver as target organs because they display a broad range of degenerative changes during normal aging that could be caused, at least in part, by transcriptional changes due to epigenetic drift. While both organs are mostly postmitotic, liver retains regenerative capacity, which would allow us to compare methylation patterns in cells that exist for a life time without replication with those that still, occasionally display proliferative activity, including polyploidization.

**Figure 1:**
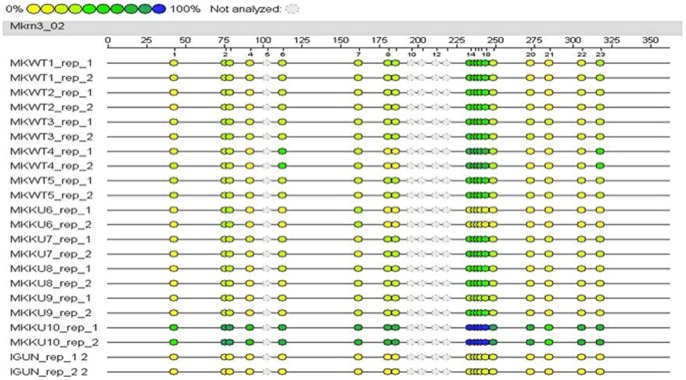
EpiGram tab for the gene Mkrn3. The EpiGram is a graphical representation of methylation ratios found in each sample for the amplicon studied. Each sample’s nucleotide sequence is displayed as a series of individual CpGs, which are color-coded columns on the same line. The color within the column denotes the level of methylation found at this particular site in the selected sample. The color spectrum ranges from yellow (or 0% methylated) CpG units to blue (100% methylated) CpG units. Grey dots denote not analyzable CpG sites. Above an example of an EpiGram obtained for the studied imprinted gene Mkrn3. The first 10 lines are relative to the five WT samples (in duplicates), lines 11 to 20 are relative to the Ku80^−/−^ samples. The last line corresponds to the unmethylated sample, used as a conversion control. Some CpG sites (i.e. CpG sites 2–3, 8–9, 14–18) were analyzed as “units” (i.e. ≥1 CpG site) because of their close vicinity to each other in the sequence.

Table 1 lists the ten genes studied for DNA methylation in their promoter region. We selected 7 imprinted genes, as well as five non-imprinted genes for comparison. For the latter we tested both cerebral cortex and liver and used some genes for which a progressive rise in DNA methylation levels across the lifespan has been observed in human *(MGMT, GAD1, HOXA1)* [19] and mouse (H1ln) brain, as well as Cradd, whose DNA methylation level has been reported to progressively decrease in mouse liver during aging ( [20]). We tested for preservation of DNA methylation at gene promoter regions in both liver and cerebral cortex using the quantitative Epityper assay. In this assay genomic DNA is first treated with bisulfite to convert non-methylated cytosine into uracil. PCR amplification of the target sequence changes the uracil into thymine and also introduces a T7 promoter tag. The latter is used for *in vitro* RNA transcription followed by cleavage at uracil bases. The cleavage products are subsequently resolved by MALDI-TOF mass spectrometric analysis into signal pairs from the methylated and non-methylated template. Using the Epityper assay changes in methylation of approximately *5*% can still be detected (ref. [18] and unpublished results). An example of a graphical representation of the CpG sites within the selected region of the imprinted *Mkrn3* promoter is shown in Fig. 1.

**Table 1:** List of gene promoters studied.

Gene	Type	Promoter Location (Chr, accession number, sequence coordinates)	Organ studied
Igf2	Imprinted	CHR7 NT_039437.8 (915766; 915522)	Liver
Mkrn3	Imprinted	CHR7 NT_187035.1 (23318842;23319183)	Liver
Pon3	Imprinted	CHR6 NT_039340.7 (2207612; 2207380)	Liver
Cradd	Not Imprinted	CHR10 gb|AE016773.1| (148267–148659)	Liver
Copg2	Imprinted	CHR6 NT_039353.8 (94313;93958)	Brain (cortex)
Nap1l5	Imprinted	CHR6 NT_039353.8 (28104222; 28104521)	Brain (cortex)
Ube3a	Imprinted	CHR7 NT_187035.1 (20128088; 20127774)	Brain (cortex)
Gad1	Not imprinted	CHR2 NT_039207.8 (11441270–11441591)	Brain (cortex)
Hoxa1a	Not imprinted	CHR6 NT_039353.8 (21355364–21355752)	Brain (cortex)
Mgmt	Not imprinted	CHR7 NT_039433.8 (54643008–54643382)	Brain (cortex)
H1ln	Not imprinted	CHR17 gb|JN963759.1| (5167–5486)	Brain (cortex)

**Figure 2:**
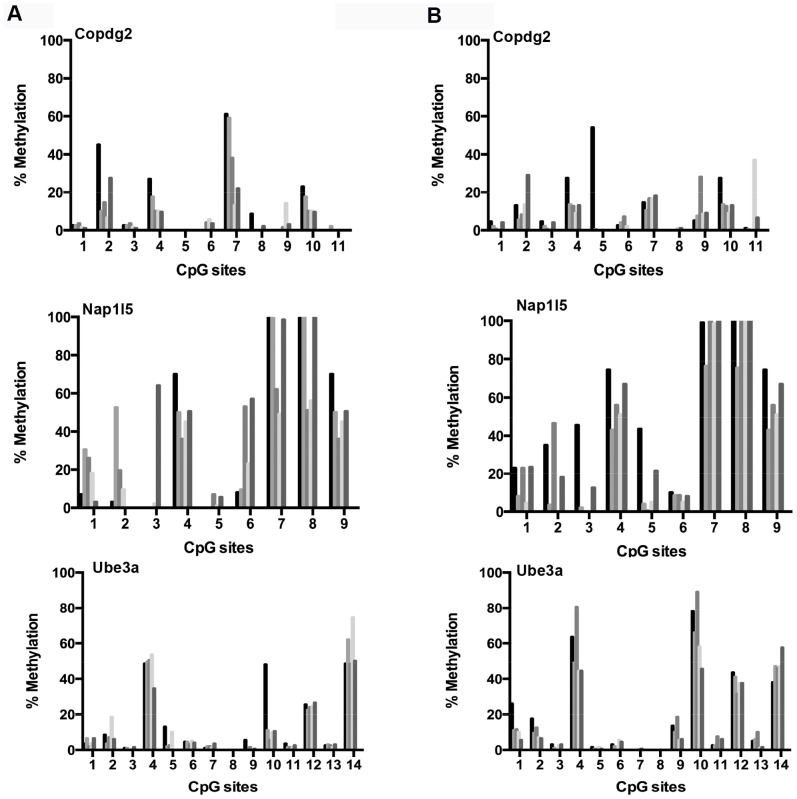
Age-related DNA methylation patterns at imprinted gene promoters in cerebral cortex. DNA methylation status in cerebral specific CpG sites within promoters of imprinted genes in young i.e., 6 months (a) and old i.e., 27 months (b) C57BL/6 mice. Each tickmark represents specific CpG sites within the imprinted promoter and each column represents the methylation status for each animal.

In aged mice, both cerebral cortex and liver are characterized by accumulated changes that could have been driven by epigenetic drift. While a number of studies have examined methylation in aging, there is still no consensus on the magnitude of the effects, particularly at imprinted loci. Fig. 2 shows a comparison of the methylation level at 34 distinct CpG sites in promoter sequences of three genes imprinted in the cerebral cortex of young (i.e., 6 months;Fig. 2a) and old (i.e., 27 months; Fig. 2b) mice. The results show that while methylation level at some of these sites varies substantially from animal to animal, their overall pattern is highly preserved. The exception was one CpG site in the *Ube3a* gene (CpG 10), which exhibited higher levels of methylation in aged animals. However, after Bonferroni correction for multiple testing this single change appeared not to be statistically significant. We performed an additional statistical comparison to test whether average levels of DNA methylation for each gene were significally different. While we found Ube3a exhibiting differential global methylation, this was not significant after correction for multiple testing (results not shown).

**Figure 3:**
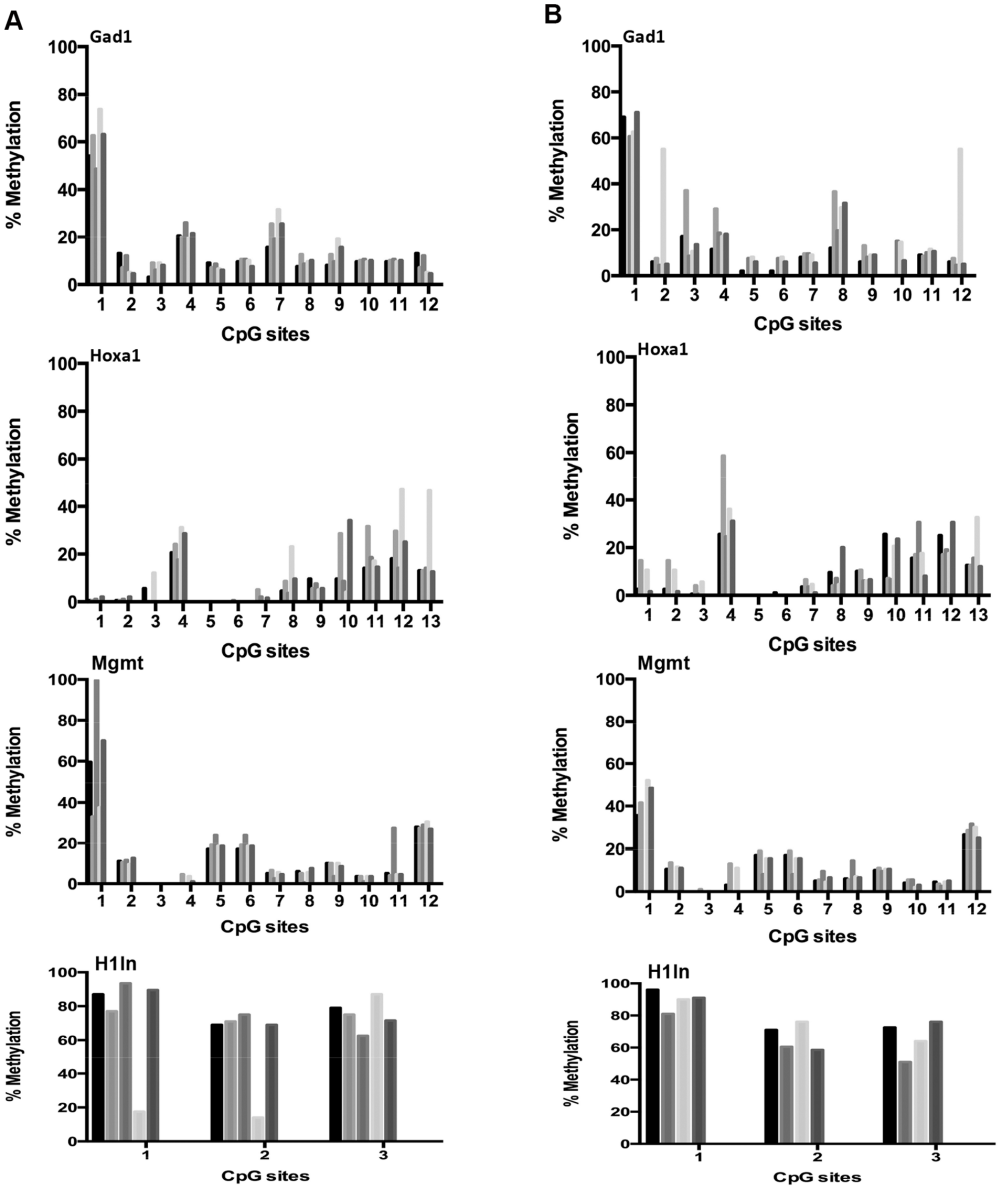
Age-related DNA methylation patterns at non-imprinted gene promoters in cerebral cortex. DNA methylation status in cerebral cortex at specific CpG sites within promoters of non-imprinted genes of young, i.e. 6 months (a), and old, i.e. 27 months (b), C57BL/6 mice. Each tickmark represents specific CpG sites within the non-imprinted promoter and each column represents the methylation status for each animal.

For comparison, we subsequently tested four non-imprinted genes and the results show that all 39 CpG sites analyzed in the promoter regions of these genes had almost exactly the same level of methylation in the young (Fig. 3a) and old (Fig. 3b) mice. Hence, these results indicate a similarly highly preserved pattern of DNA methylation as observed in the promoter regions of imprinted genes.

**Figure 4:**
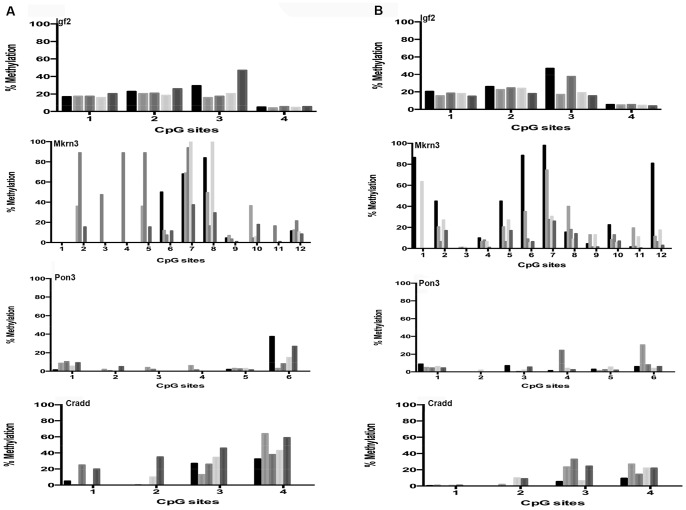
Age-related DNA methylation patterns at imprinted and non-imprinted gene promoters in liver. DNA methylation status in liver at specific CpG sites within promoters of imprinted genes of young, i.e. 6 months (a) and old, i.e. 27 months (b) C57BL/6 mice liver. Each tickmark represents specific CpG sites within the imprinted promoter and each column represents the methylation status for each animal.

We subsequently analyzed mouse liver for age-related changes in gene promoter methylation at 22 CpG sites in 3 genes that are imprinted in this organ, chosen from the database for imprinted genes (http://igc.otago.ac.nz/home.html). The results were very similar to what was observed in cerebral cortex, i.e., a very similar quantitative pattern in the young (Fig. 4a) and old (Fig. 4b) animals. For *Mkrn3* we observed, both in young and old mice, an unexplained high animal-to-animal variation, which was significant when compared to the other tested genes (p = 0.004, 0.034 and 0.042 for *Pon3*, *Igf2* respectively). Similarly to what was done for cerebral cortex, for comparison we tested a non-imprinted gene (*Cradd*) and we found, in keeping with a previous report [20], lower levels of DNA methylation associated with aging at a particular CpG site (CpG 4; Fig. 4a,b, bottom). However, after Bonferroni correction for multiple testing this single change appeared not to be statistically significant. Additionally, comparison of average DNA methylation levels for the *Cradd* gene indicates an age-related decline in methylation (p = 0.03, see Table S6 in File S1).

### DNA Methylation Analysis in Prematurely Aged, DNA Repair Defective Mice

DNA damage has been implicated as a major causal factor in aging, which is in keeping with the observation that genetic defects in DNA repair often lead to segmental premature aging in both humans and mice [21]. DNA damage and repair may lead to heritable loss of methylation at some sites, for example, because of increased cell turnover during regeneration or elaborate translesion DNA synthesis during replication. There is evidence that DNA methylation patterns are affected by DNA damage and repair. For example, restoration of cytosine methylation patterns after DNA excision repair is incomplete [22] and *de novo* methylation has been found associated with homology-directed repair of DNA double-strand breaks, which involves extensive DNA resynthesis [23]. Replication and repair in the context of chromatin is a major challenge [24] and it is conceivable that DNA repair defects would impair propagation of DNA methylation patterns. Hence, we assessed stability of DNA methylation in promoter regions of imprinted genes in mice harboring specific defects in DNA repair concurrent with multiple symptoms of premature aging.

The two distinct prematurely aging models included in this study were harboring defects in *Ercc−/d7* and *Ku80*, respectively. The *Ercc−/d7* mouse model carries a null mutation in one allele and a 7-amino acid C-terminal truncation in the second allele, which still permits a partial repair function. These animals show deficiencies in nucleotide excision repair, interstrand crosslink repair and homologous recombination dependent DNA double-strand break repair and usually die from dramatic aging-like changes causing severe liver and kidney failure around 6 months [25]. Additionally, these mice display numerous other progeroid features, including accelerated aging-associated changes in the bone marrow, skeleton, and the neurological and cardio-vascular systems. The *Ku80* mutant mice harbor two null alleles and are defective in the repair of DNA double-strand breaks through non-homologous endjoining (NHEJ). These animals have a maximum life span of 14 months and also suffer from premature liver aging [26], kyphosis and skeletal alterations but have otherwise a milder aging-associated phenotype besides increased cancer predisposition. Both mouse models were kept in a C57BL/6-FVB background. The 50% FVB background was necessary because C57BL/6 alone is embryonically lethal for both the *Ercc−/d7* and the *Ku80*-deficient genotype.

**Figure 5:**
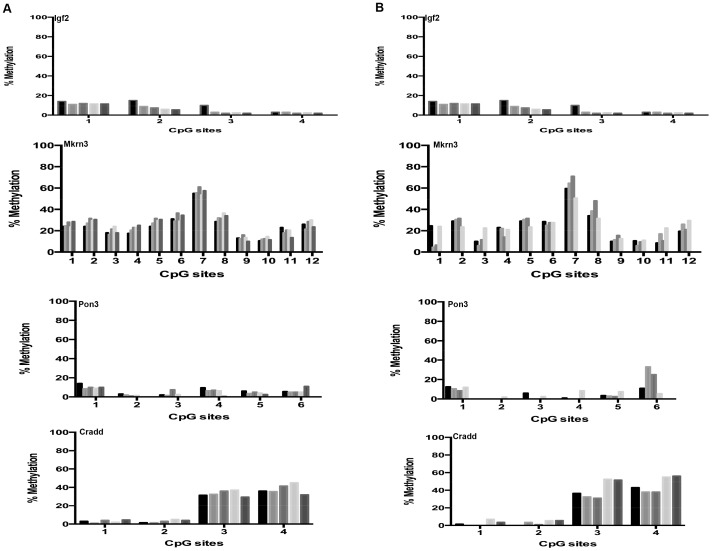
DNA methylation patterns at imprinted and non-imprinted gene promoters in *Ercc-/d7* mutant mice. DNA methylation status in liver at specific CpG sites within promoters of imprinted genes of *WT* (a) and *Ercc-/d7* (b) mice liver (both 14 weeks of age). Each tickmark represents specific CpG sites within the imprinted promoter and each column represents the methylation status for each animal.

**Figure 6:**
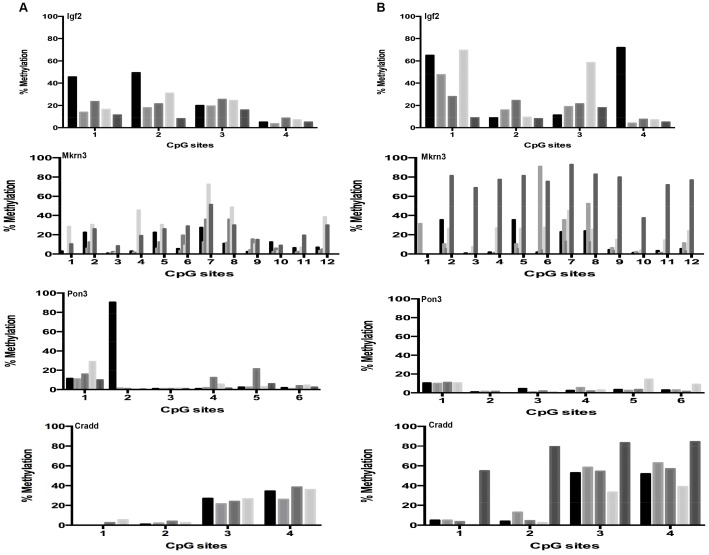
DNA methylation patterns at imprinted and non-imprinted gene promoters in *Ku80^−/−^* mutant mice. DNA methylation status in liver at specific CpG sites within promoters of imprinted genes of *WT* (a) and *Ku80^−/−^* (b) mice liver (both 10 months of age). Each tickmark represents specific CpG sites within the imprinted promoter and each column represents the methylation status for each animal.

Because liver is the organ most severely affected by premature aging in both DNA repair defective models we analyzed site-specific DNA methylation only in liver. DNA was extracted from 14 weeks Ercc*−/d7* and 10-month old *Ku80* mutant mice. Figs. 5 and 6 show DNA methylation levels for *Ercc−/d7* and *Ku80* mutant mice at in total 24 CpG sites, indicating an overall similar conservation of CpG-specific modification patterns as observed during normal aging with the exception of the non-imprinted gene, Cradd, which showed an increase in DNA methylation in the Ku80 mutant mice (at CpG site 4; Fig.6, bottom). However, after Bonferroni correction for multiple testing this single change appeared not to be statistically significant. While CpG-specific cytosine methylation in *Ku80* mutant liver shows a high individual variation, particularly for *Mkrn3*, when tested for homogeneity of variance this difference in individual variation appeared not to be significant.

## Discussion

There are two types of DNA methylation changes that could occur during aging [27]. First, concerted hypo-or hyper-methylation at specific CpG sites could occur systematically among the various cells in an organ or tissue in response to environmental or endogenous stresses, such as genotoxic stress, protein aggregation and inflammation. On the other hand, changes in DNA methylation could be stochastic and merely occur as a consequence of incomplete restoration of methylation patterns, for example, after repair of DNA damage [22], or hypermethylation due to overactive DNA methyltransferase [28]. Here we specifically analyzed individual CpG sites (in total 112) in seven imprinted genes in liver and cerebral cortex from young (6 months) and old mice (27 months) and in four non-imprinted genes in cerebral cortex and one in the liver. Although for some genes we noted substantial inter-individual variation the results indicate that overall patterns of CpG methylation are remarkably well preserved during the aging process. Defects in major DNA repair processes, which would presumably erode DNA methylation maintenance, made no difference and still did not lead to significant alterations in cytosine methylation.

Since the limit of detection of the method used to quantify DNA methylation at individual CpG sites is approximately 5%, our results do not rule out much smaller changes. However, stochastic changes in the epigenome, i.e., epimutations, are likely to occur at a rate that is 2–3 orders of magnitude higher than mutation rate. Indeed, maintenance methylation efficiency has been estimated at ∼96% per cell division [29]. Based on these estimates it would not have been surprising to find considerable changes during aging at virtually all CpG sites. Clearly, this is not the case. By contrast, the high plasticity of the DNA methylome in cancer has been well documented [30] and must be ascribed to the combination of selection for high genome instability and expression changes, due to high replicative stress and frequent loss of one or more genome maintenance processes and the high proliferation rate as compared to aging in liver and especially cerebral cortex.

In conclusion, this semi-quantitative analysis of multiple specific CpG sites in promoter regions of several randomly chosen imprinted genes highlights that, despite inter-individual variability, DNA methylation patterns are remarkably stable. Our findings suggest that the tested somatic tissues have an adequate DNA methylation maintenance system at their disposal to maintain imprinted loci over time.

## Supporting Information

File S1 (DOC)
