# Supplementary material for: High Preservation of CpG Cytosine Methylation Patterns at Imprinted Gene Loci in Liver and Brain of Aged Mice
Source: PLoS One. 2013 Sep 9;8(9):e73496. doi: 10.1371/journal.pone.0073496 (PMC3767788; doi:10.1371/journal.pone.0073496)
Supplement: File S1 — (DOC) [file pone.0073496.s001.doc]

**Table S1 Pvalues of DNA mehtylation levels of individual CpG sites in brain of young vs. old mice**

| **Brain**  **(Young versus Old)** | **pval** | **Brain**  **(Young versus Old)** | **pval** |
| --- | --- | --- | --- |
| Copdg2.1 | 1 | Gad 1.9 | 0.28571429 |
| Copdg2.2 | 0.547619048 | Gad 1.10 | 1 |
| Copdg2.3 | 1 | Gad 1.11 | 0.412 |
| Copdg2.4 | 0.83403523 | Gad 1.12 | 0.58143314 |
| Copdg2.5 | 0.240420786 | Hoxa.1 | 0.171904309 |
| Copdg2.6 | 0.901704804 | Hoxa.2 | 0.171904309 |
| Copdg2.7 | 0.093692619 | Hoxa.3 | 0.828262541 |
| Copdg2.8 | 0.723058127 | Hoxa.4 | 0.116073943 |
| Copdg2.9 | 0.19047619 | Hoxa.5 | NA |
| Copdg2.10 | 0.83403523 | Hoxa.6 | 1 |
| Copdg2.11 | 0.118797498 | Hoxa.7 | 0.20729984 |
| Napl15.1 | 1 | Hoxa.8 | 1 |
| Napl15.2 | 0.916562645 | Hoxa.9 | 0.20729984 |
| Napl15.3 | 0.737315677 | Hoxa.10 | 0.841269841 |
| Napl15.4 | 0.30952381 | Hoxa.11 | 0.916562645 |
| Napl15.5 | 0.294801992 | Hoxa.12 | 0.753298033 |
| Napl15.6 | 0.114961001 | Hoxa.13 | 0.52451828 |
| Napl15.7 | 0.588809187 | Mgmt.2 | 1 |
| Napl15.8 | 0.440686016 | Mgmt.3 | 0.423710797 |
| Napl15.9 | 0.30952381 | Mgmt.4 | 0.56136321 |
| Napl15.9 | 0.30952381 | Mgmt.5 | 0.090688366 |
| UBE3a.1 | 0.035578833 | Mgmt.6 | 0.090688366 |
| UBE3a.2 | 0.463343883 | Mgmt.7 | 0.397614752 |
| UBE3a.3 | 0.241844304 | Mgmt.8 | 0.20729984 |
| UBE3a.4 | 0.83403523 | Mgmt.9 | 0.104870291 |
| UBE3a.5 | 0.137563894 | Mgmt.10 | 0.387093697 |
| UBE3a.6 | 0.457807394 | Mgmt.11 | 0.33054711 |
| UBE3a.7 | 0.010662275 | Mgmt.12 | 1 |
| UBE3a.8 | NA | Hn1l.1 | 0.28571429 |
| UBE3a.9 | 0.01565054 | Hn1l.2 | 1 |
| UBE3a.10 | 0.015873016 | Hn1l.3 | 0.412 |
| UBE3a.11 | 0.391667792 |  | |
| UBE3a.12 | 0.011925234 |  | |
| UBE3a.13 | 0.598161453 |  | |
| UBE3a.14 | 0.05855263 |  | |
| UBE3a.14 | 0.05855263 |  | |
| Gad1.1 | 0.137563894 |  | |
| Gad1.2 | 0.59928633 |  | |
| Gad 1.3 | 0.675173615 |  | |
| Gad 1.4 | 0.675173615 |  | |
| Gad 1.5 | 0.208690443 |  | |
| Gad 1.6 | 1 |  | |
| Gad 1.7 | 1 |  | |
| GAD1.8 | 1 |  | |

1

| **Liver**  **(Young versus Old)** | **pval** |
| --- | --- |
| Mkrn3.1 | 0.179712495 |
| Mkrn3.2 | 0.571428571 |
| Mkrn3.3 | 0.68122512 |
| Mkrn3.4 | 0.260174901 |
| Mkrn3.5 | 0.571428571 |
| Mkrn3.6 | 0.73015873 |
| Mkrn3.7 | 0.30952381 |
| Mkrn3.8 | 0.055555556 |
| Mkrn3.9 | 0.619796388 |
| Mkrn3.10 | 0.600401848 |
| Mkrn3.11 | 0.138791738 |
| Mkrn3.12 | 0.916562645 |
| Pon3.1 | 0.401965358 |
| Pon3.2 | 0.287725739 |
| Pon3.3 | 0.452862183 |
| Pon3.4 | 0.279641534 |
| Pon3.5 | 0.914999049 |
| Pon3.6 | 0.461975431 |
| Pon3.6 | 0.461975431 |
| Igf2.1 | 1 |
| Igf2.2 | 0.600401848 |
| Igf2.3 | 0.916562645 |
| Igf2.4 | 1 |
| Cradd.1 | 0.91390646 |
| Cradd.2 | 0.15079365 |
| Cradd.3 | 0.01192523 |
| Cradd.4 | 0.01192523 |

**Table S2 Table 1 Pvalues of DNA mehtylation levels of individual CpG sites in liver of young vs. old mice**

**Table S3 Pvalues of DNA mehtylation levels of individual CpG sites in liver of**

**WT versus Ercc-/d7 mice)**

**Table S4 Pvalues of DNA mehtylation levels of individual CpG sites in liver of**

**WT versus Ku80 KO mice**

| **Liver**  **WT versus Ercc-/d7** | **pval** |
| --- | --- |
| Mkrn3.1 | 0.174277021 |
| Mkrn3.2 | 1 |
| Mkrn3.3 | 0.176097771 |
| Mkrn3.4 | 0.533388948 |
| Mkrn3.5 | 1 |
| Mkrn3.6 | 0.018942894 |
| Mkrn3.7 | 0.412698413 |
| Mkrn3.8 | 0.266322041 |
| Mkrn3.9 | 0.536878456 |
| Mkrn3.10 | 0.049090116 |
| Mkrn3.11 | 0.268340589 |
| Mkrn3.12 | 0.460558169 |
| Pon3.1 | 0.621283452 |
| Pon3.2 | 0.247455063 |
| Pon3.3 | 0.80241931 |
| Pon3.4 | 0.266322041 |
| Pon3.5 | 0.710991996 |
| Pon3.6 | 0.059451093 |
| Pon3.6 | 0.059451093 |
| Igf2.1 | 0.747262026 |
| Igf2.2 | 0.114961001 |
| Igf2.3 | 0.385546632 |
| Igf2.4 | 0.44319355 |
| Igf2.5 | 0.44319355 |
| Cradd.1 | 0.52961930 |
| Cradd.2 | 1 |
| Cradd.3 | 0.34574183 |
| Cradd.4 | 0.14245670 |

| **Liver**  **(WT versus Ku80)** | **pval** |
| --- | --- |
| Mkrn3.1 | 0.687321801 |
| Mkrn3.2 | 0.69047619 |
| Mkrn3.3 | 0.672335808 |
| Mkrn3.4 | 0.901704804 |
| Mkrn3.5 | 0.69047619 |
| Mkrn3.6 | 0.69047619 |
| Mkrn3.7 | 1 |
| Mkrn3.8 | 0.547619048 |
| Mkrn3.9 | 1 |
| Mkrn3.10 | 0.345741826 |
| Mkrn3.11 | 0.83403523 |
| Mkrn3.12 | 1 |
| Pon3.1 | 0.172447345 |
| Pon3.2 | 1 |
| Pon3.3 | 0.308369336 |
| Pon3.4 | 0.710991996 |
| Pon3.5 | 0.900437537 |
| Pon3.6 | 0.712154538 |
| Pon3.6 | 0.712154538 |
| Igf2.1 | 0.222222222 |
| Igf2.2 | 0.249152639 |
| Igf2.3 | 0.69047619 |
| Igf2.4 | 0.595883091 |
| Igf2.5 | 0.595883091 |
| Cradd.1 | 0.26632204 |
| Cradd.2 | 0.06393675 |
| Cradd.3 | 0.01587302 |
| Cradd.4 | 0.01587302 |

| Sample  (brain) | **Gene/mean value** | | | | | | |  |
| --- | --- | --- | --- | --- | --- | --- | --- | --- |
|  | Copdg2 | | | Nap1l5 | | Ube3a | |  |
| Y1 | 18.8333333 | | | 39.7777778 | | 15.25 | |  |
| Y2 | 11.1 | | | 43.6111111 | | 12.0714286 | |  |
| Y3 | 7.7272727 | | | 32.2777778 | | 10.75 | |  |
| Y4 | 5.6363636 | | | 27.6111111 | | 14.6071429 | |  |
| Y5 | 7.1818182 | | | 47.6666667 | | 10.6428571 | |  |
| O1 | 14 | | | 56.1111111 | | 21.0714286 | |  |
| O2 | 5.4090909 | | | 29.3333333 | | 17.5357143 | |  |
| O3 | 7.7727273 | | | 43.3888889 | | 21.9642857 | |  |
| O4 | 8.8636364 | | | 35 | | 15.75 | |  |
| O5 | 8.8636364 | | | 46.3888889 | | 15.6428571 | |  |
| PValue | 0.698 | | | 0.5333 | | 0.0094 | |  |
| **Sample**  **(brain)** | | **Gene/mean value** | | | | | | |
|  | | Gad1 | Hoxa1 | | Mgmt | | H1ln | |
| Y1 | | 14.3333333 | 7.3461538 | | 13.5 | | 78.33333333 | |
| Y2 | | 16.125 | 11.2692308 | | 11.875 | | 74.33333333 | |
| Y3 | | 15.125 | 6.7692308 | | 20.2727273 | | 77 | |
| Y4 | | 17.2916667 | 14.2692308 | | 12.25 | | 39.5 | |
| Y5 | | 15.5 | 10.3846154 | | 14.6666667 | | 76.66666667 | |
| O1 | | 12.9583333 | 10.2692308 | | 11.5833333 | | 79.83333333 | |
| O2 | | 14.2727273 | 12.7692308 | | 13.6666667 | | 64.16666667 | |
| O3 | | 14.6666667 | 8.6923077 | | 8.9 | | NA | |
| O4 | | 24.4166667 | 12.7307692 | | 13.6666667 | | 76.66666667 | |
| O5 | | 16 | 10.4230769 | | 12.25 | | 75.16666667 | |
| PValue | | 0.7251 | 0.5602686 | | 0.21 | | 0.58143314 | |

**Table S5 Average percentage of DNA methylation in the brain (young versus old)**

| **Sample**  **(liver)** | **Gene/mean value** | | | |
| --- | --- | --- | --- | --- |
|  | Mkrn3 | Pon3 | Igf2 | Cradd |
| Y1 | 21.8 | 6.833333333 | 18.625 | 16.125 |
| Y2 | 21.54166667 | 4.416666667 | 14.5 | 19.5 |
| Y3 | 39.875 | 4.166666667 | 15.375 | 22.25 |
| Y4 | 36 | 3.916666667 | 14.875 | 22 |
| Y5 | 11.5 | 8.6 | 24.75 | 40 |
| O1 | 41.5 | 4.416666667 | 24.75 | 3.875 |
| O2 | 20.875 | 6.166666667 | 15 | 13.375 |
| O3 | 8.291666667 | 6.666666667 | 21.5 | 11.875 |
| O4 | 18.08333333 | 3.916666667 | 16.375 | 9.875 |
| O5 | 7.791666667 | 3.416666667 | 13.125 | 14.125 |
| PValue | 0.420 | 0.567 | 0.861 | 0.02878204 |

**Table S6 Average percentage of DNA methylation in liver (young versus old)**

**Table S7 Average percentage of DNA methylation in liver (WT versus Ercc-/d7)**

| **Sample** | **Gene/mean value** | | | |
| --- | --- | --- | --- | --- |
|  | **Mkrn3** | **Pon3** | **Igf2** | **Cradd** |
| **WT1** | 23.83333333 | 5.666666667 | 6.125 | 18 |
| **WT2** | 23.5 | 7.75 | 5.375 | 17.625 |
| **WT3** | 24.83333333 | 6 | 4.875 | 21.125 |
| **WT4** | 24.95833333 | 6.333333333 | 5.625 | 22.25 |
| **WT5** | NA | NA | 5.125 | 17.5 |
| **Ercc-/d7-1** | 24.54166667 | 6.666666667 | 10.5 | 20.25 |
| **Ercc-/d7** -**2** | 24.66666667 | 4.5 | 6.5 | 18.5 |
| **Ercc-/d7-** **3** | 28.5 | 5.916666667 | 5.875 | 17.5 |
| **Ercc-/d7** -**4** | 27.25 | 4.75 | 5.625 | 30 |
| **Ercc-/d7** -**5** | 26.41666667 | 4 | 5.25 | 29.125 |
| **PValue** | 0.0581 | 0.100 | 0.861 | 0.24417721 |

**Table S8 Average percentage of DNA methylation in liver (WT versus Ku80 KO)**

| **Sample** | **Gene/mean value** | | | |
| --- | --- | --- | --- | --- |
|  | Mkrn3 | Pon3 | Igf2 | Cradd |
| WT1 | 10.375 | 18.08333333 | 30 | 15.625 |
| WT2 | 4.625 | 3.166666667 | 13.75 | 12.375 |
| WT3 | 12.45833333 | 9.333333333 | 19.75 | 17.25 |
| WT4 | 27.16666667 | 7.333333333 | 19.75 | 17.625 |
| WT5 | 22.83333333 | 3.583333333 | 10.125 | NA |
| Ku80KO-1 | 11.5 | 4.166666667 | 39.375 | 28.5 |
| Ku80KO-2 | 23.04545455 | 3.916666667 | 21.625 | 34.875 |
| Ku80KO-3 | 4.291666667 | 3.583333333 | 20.375 | 29.875 |
| Ku80KO-4 | 20.25 | 6.333333333 | 36.125 | 18.875 |
| Ku80KO-5 | 75.22727273 | NA | 10 | 75.625 |
| PValue | 0.430 | 0.236 | 0.322 | 0.09104188 |

**Fig.S1**

A representative example of an Epipanel design for the Gad1 gene. The diagram provide information about individual CpG methylation (upper panel), location of the amplification targets (middle panel; in our case, we selected the Gad_01), and gene structure (lower panel) within context of genomic annotation. Figure was adapted from the mouse epi-panel, available at www.sequenom.com/getdoc/913a6da8.../mouse_epipanel
